# Sequential Effects in SNARC

**DOI:** 10.1038/s41598-018-29337-2

**Published:** 2018-07-20

**Authors:** Dinis Gökaydin, Peter Brugger, Tobias Loetscher

**Affiliations:** 1 School of Psychology, Social Work and Social Policy, University of South Australia, Adelaide, Australia; 2 Department of Neurology, University Hospital Zürich, Zürich, Switzerland

## Abstract

Small and large numbers are typically associated with the left and right side of space, respectively. We conducted an online version of the classical Spatial-Numerical Association of Response Codes (SNARC) paradigm in 604 subjects in order to analyse how previous trials and responses affect SNARC. Our results point to a strong inversion of number-space associations (left/large and right/small) when the last trial was incoherent - i.e. when a response with the left hand was made to a large number or vice-versa. In addition, we demonstrate that the congruency of trials beyond just the last two can influence SNARC, providing empirical support for an important assumption of a working memory account of spatial-numerical associations. Finally, we show that sequential effects in SNARC can be captured by a simple exponential filter, known to underpin sequential effects across a range of stimuli detection and perceptual two-alternative forced choice decision tasks. Our findings point to universal mechanisms responsible for the processing of sequences from perception to cognition.

## Introduction

People with a western background tend to respond faster to small numbers with the left hand and quicker to higher numbers with the right hand. This is traditionally interpreted as evidence for an association between number magnitude and a left-right axis, or ‘mental number line’. The effect was first described by Dehaene *et al*.^1^ who coined the term Spatial-Numerical Association of Response Codes (SNARC). Several factors are known to influence the SNARC effect such as reading direction^2^, working memory demands^3,4^, positioning of numbers^5^, instructions^6^, as well as the participants’ neuropsychological status, especially concerning the presence of deficits in spatial attention^7^. These factors not only influence the magnitude of the SNARC effect but, in some circumstances, can also invert it so that subjects respond quicker to small numbers with the right hand and vice-versa. Such an inversion of the SNARC effect was observed, for example, in Palestinians who read and write numbers from right to left^2^. Similarly, an inversion of the SNARC effect was also observed when subjects were instructed to imagine numbers positioned around a clock face^6^.

The malleability of the SNARC effects extends even further as it has been found to depend on a trial-by-trial basis on the pattern of previous single digit stimuli: Tan *et al*.^8^ found that the effect was present after stimulus alternations (e.g., 3 followed by 4) but not after stimulus repetitions (e.g., 3 followed by 3). Moreover, the SNARC effect was also found to depend on a trial-by-trial basis on the congruency of previous trials, where a congruent trial (C) is defined as one where a response was made with the left hand to a small number or with the right hand to a large number; conversely, an incongruent trial (I) is one where a response was made with the right hand to a small number or the left hand to a large number. Pfister *et al*.^9^ showed that the SNARC effect is attenuated after incongruent trials, but for repetitions of the same response, not for alternations.

So the SNARC effect has been found to depend on the past history of stimuli, the history of responses, and on the congruency of the previous trial. However, it is not entirely clear how these different dependencies are related. Previous studies show only isolated snippets of how the history of stimuli, responses and congruency might be related. Tan *et al*.^8^ show that SNARC depends on the last stimulus but not previous ones; Pfister *et al*.^9^ show that there is an interaction between how SNARC depends on previous responses and the congruency of previous trials, but again only for an isolated case corresponding to the last trial. However, there is an extensive literature on sequential effects pointing to the fact that looking at only the last trial can be misleading as this is usually part of a more complex dependence on all possible histories of trials going back as far as five^10^. With this in mind, one of the objectives of this work is to perform a detailed analysis of the way in which SNARC depends on the history of stimuli, responses and congruency, as well as the way in which these types of dependence on different sequences interact.

Once we have established how SNARC depends on different experimental parameters we will evaluate its significance by putting our results in context with what is known from the literature on sequential effects in two-alternative forced-choice (2AFC) tasks. Traditionally, sequential effects are studied in simple two-alternative forced-choice tasks where reaction time is measured as a function of the history of two possible stimuli. In this setting, it is thought that sequential effects are the product of expectations developed by the sequence of trials and whether it displays a pattern or not. While also ultimately based on RT measurements, SNARC is a higher-level cognitive effect, the magnitude of which is measured using the slope of the correlation between number and difference in RT between right and left responses. Demonstrating that sequential effects also occur in SNARC would therefore contribute towards a view of sequential effects as part of a universal mechanism operating all the way from lower-level perception up to higher-level cognition.

### Sequential effects

For the purposes of this discussion ‘sequential effects’ is to be understood as the dependence of any behavioural measure on all possible histories of events, as opposed to just the last two. Such studies have been most commonly conducted in the context of 2AFC tasks where reaction time (RT) forms the dependent measure^10–12^. However, sequential effects similar to those identified in RT have been observed in a multitude of different experimental measures, such as the point of subjective equality in psychophysical tasks^13,14^, event-related potentials in EEG^15–17^ and prediction probability^18^. In addition, there is evidence for the existence of two components of sequential effects: one perceptual in origin and associated with the sequence of stimuli and another of motor origin and associated with the sequence of responses^13,17,19,20^.

Sequential effects are thought to be the trace of an attempt at detecting a pattern in the sequence of events. The reason for this view is that the impact the sequence has on behaviour is strongest for - although not limited to - perfectly regular sequences, specifically repetitions of the same element or alternations of two elements. Moreover, the effect of the sequence of events decays exponentially fast, so that only the latest few trials modulate behaviour^21^. With this in mind, consider the sequences XYXY and XXXX - where X/Y denote two possible stimuli and time flows from left to right - as two possibilities for the last five stimuli a subject has seen: in both cases humans tend to respond quicker is the next stimulus is an X, and slower if it is a Y, and this is taken as evidence that people expected the pattern to continue. This illustration should not detract from the fact that any sequence of events - whether regular or not - has systematic and predictable effects on human behaviour, and as such it is crucial to look at all possible histories of events, i.e. to look at the *profile* of sequential effects across all sequences.

Different mathematical models have been proposed in order to account for the profile of sequential effects across all recent sequences of events^12,22–25^. Such models differ in their details but have one element in common: they all include in some form an exponentially weighted average of previous events, or exponential filter. The exponential filter can be explained intuitively as follows: the more one sees of a particular stimulus the greater the expectation that another of the same type will occur in the next step, and this effect decays at a constant rate - i.e. exponentially - into the past, so that stimuli observed more than a few steps into the past have a negligible impact on predictions. This exponential ‘forgetting’ of past events is a well established feature of human memory (There is some debate regarding the specific form of the forgetting function, but this has only a negligible effect on model predictions^26^). For instance, in the case where RT is the dependent measure, and denoting the current event as *x*_*t*_, the event at *x*_*t*−5_ is usually found to have little influence on results, and therefore traditionally sequential effects rely on the analysis of all possible sequences of recent events all the way back to *x*_*t*−4_^21^. In recursive form the exponential filter can be written

1 $$p({x}_{t+1})=\alpha {x}_{t}+\mathrm{(1}-\alpha )p({x}_{t})$$

where *x*_*t*_ represents the current event - coded as 0 or 1 - and *α* is a memory parameter regulating how fast the filter ‘forgets’ past events; *p*(*x*_*t*_) is the probability that the next event is equal to 1, and is usually interpreted psychologically as a subjective probability estimate of what the next event will be. High values of *α* imply a shorter ‘memory’, i.e. only the last few events will have any significant effect on the probability estimate for the next event; conversely, low values of *α* imply a longer memory and events further back can influence probability estimates. When fitting empirical data, model predictions will be transformed according to *b *+ *ap*(*x*_*t*_), for a total of three free parameters in the model: *a*, *b* and *α*.

The probability attributed to the next element in the sequence - say X - by the exponential filter is a function of how many times, as well as how recently, that element occurred. As such the exponential filter is sensitive to repetitions of the same stimulus but insensitive to alternations thereof. Take the sequence XYXY for instance: applying the exponential filter to such a sequence results in *p*(*x*_*t*+1_ = *Y*) > *p*(*x*_*t*+1_ = *X*) since the two Y’s in the sequence were observed more recently than the X’s, despite the fact that the sequence is clearly alternating. However, in many circumstances humans not only display a sensitivity to alternations, but can even be more sensitive to alternations than repetitions^11,20^. In order to model this sensitivity to alternations several models include another type of exponential filter, similar in every respect to that in Equation (1) but applied to a binary sequence where each event represents either a repetition or alternation of the primary stimuli^22,24^. The simple sum of the predictions of both types of filter provides a very god fit to the results of some RT experiments^24^. Pragmatically however we did not encounter any sensitivity to alternations in our results and so we will use only the simple exponential filter - applied to the sequence of primary stimuli - throughout. The possible reasons behind this lack of sensitivity to alternations are discussed below.

## Method

The experiment was performed online and hosted using Google App Engine (GAE). The fundamental details of the experiment were coded in Javascript, with html/css used to design the website’s look.

### Participants

604 subjects (280 female, 324 male) participated in the experiments. Subjects were recruited using Amazon’s Mechanical Turk (AMT) system. Only participants with at least 500 tasks completed and with an approval rate of 95% or above were accepted. After accepting the task participants were directed to an externally hosted website where they had to complete the experiment. Upon completing the experiment participants were given a code which they had to insert in AMT’s website. All participants gave their informed consent to taking part in the experiment and were paid 2.1 USD for their participation. This research project has been approved by the Flinders University Social and Behavioural Research Ethics Committee and the experiment was performed in accordance with relevant guidelines and regulations.

### Stimuli

Stimuli consisted of the set of numbers 1–4 and 6–9 displayed in the middle of the browser window. The numbers were black and displayed against a gray (RGB 0.5/0.5/0.5) background.

### Procedure

The experiment was a two-alternative forced choice task (2AFC) where subjects had to identify the parity (odd/even) of the number being displayed. Each trial started with a single number being displayed; once the subject responded the number disappeared and, after a fixed response-stimulus interval (RSI) of 1000 ms, another number appeared. Subjects were instructed to respond as quickly and as accurately as possible to each number. The experiment consisted of 320 trials divided into two blocks of 160 trials each. Each block consisted of the set of numbers 1–4 and 6–9 repeated 20 times and randomly permuted; this way the frequency of each number within each block, and for the whole experiment, was equal.

Responses were made by pressing either the ‘f’ of the ‘j’ key. The mapping of the keys to number parity was different for each of the two blocks of trials. So if during the first block subjects used ‘f’ to respond ‘odd’ and ‘j’ to respond ‘even’, the assignment was reversed for the second block. The mapping for the first block was assigned randomly. Responses with any other key were not allowed, and generated an erro message warning participants to only use the ‘f’ and ‘j’ keys.

### Data analysis

**Figure 1:**
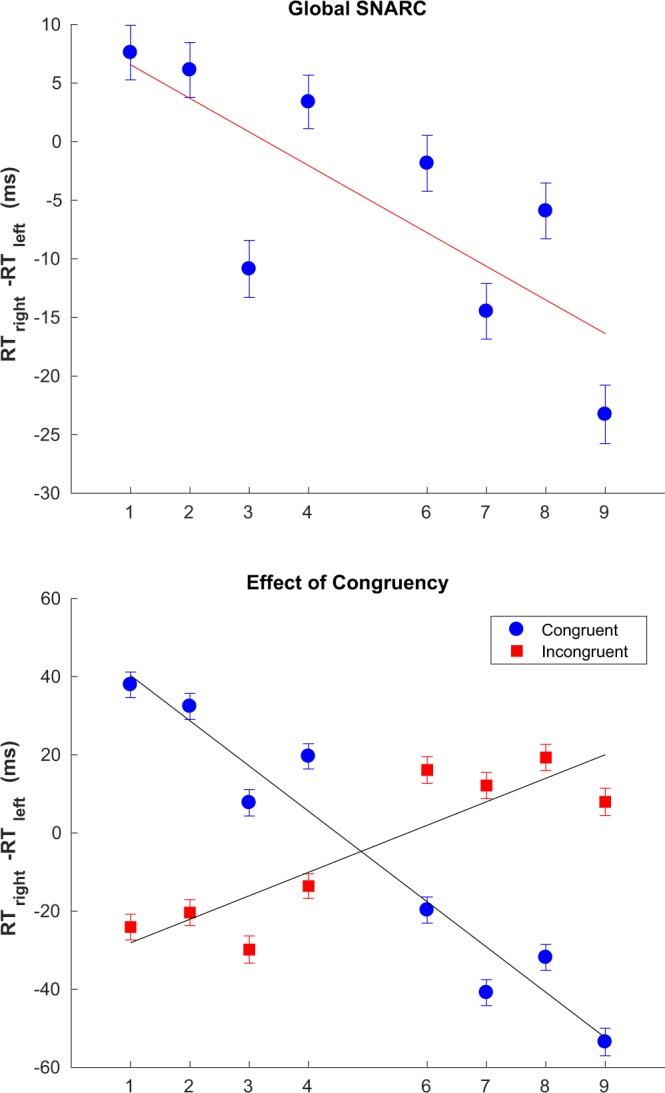
Global SNARC effect (top) and the impact of the congruency of last trial (bottom). Best fitting parameters: global SNARC *b* = 9.42, *a* = −2.86 (*CI*_*α*=0.05_ = [−4.73, −0.99]); incongruent last trial *b* = −34.13, *a* = 6.01 (*CI*_*α*=0.05_ = [3.45,8.57]); congruent last trial: *b* = 51.88, *a* = −11.58 (*CI*_*α*=0.05_ = [−13.95, −9.21]). Error bars show the standard error of the difference between means.

This work relies on reaction times collected online, instead of the more traditional way of collecting reaction times in the lab, sometimes through the use of highly accurate response boxes. In contrast, reaction times collected via a web browser can be noisier due to the use of different operating systems, web browsers and keyboards. For this reason, it was important to reproduce two hallmark results before proceeding, namely: SNARC, i.e. the association between high/low numbers and the use of the right/left hand to respond; and the typical profile of sequential effects usually observed in 2AFCs with a relatively long response-stimulus interval. In the former case Fig. 1 (top panel) clearly shows SNARC to have been successfully reproduced. With respect to sequential effects, a profile similar in every respect to traditional results was obtained (not shown). Moreover, several classic reaction time results such as Stroop and Simon effects as well Posner cueing tasks have been replicated online, further validating our approach^27^.

The magnitude of the SNARC effect was calculated as the slope of the best fitting line (*b* + *ax*) to the the difference between the mean RT with the right hand and the mean RT with the left hand, as a function of the number being responded to (belonging to the set {1, 2, 3, 4, 6, 7, 8, 9}). This analysis was performed for all trials and all subjects pooled together, or for those subsets of trials relevant in each case, for instance those trials in which the previous trial was congruent and in which a response repetition was made. Trials in which a mistake was made were excluded from the analysis and values outside 2.5 standard deviations of the log transformed RTs were excluded as outliers^28^. The significance of the parameter estimates for coefficient *a* - the slope - was calculated by using the standard error of the parameter fit assuming normally distributed slope values. Table 1 shows detailed results for the main SNARC effect shown in Fig. 1 (top panel).

**Table 1:** Summary data for the main SNARC effect.

Digit	1	2	3	4	6	7	8	9
Total number of trials	22754	22743	22691	23108	22861	22893	22898	22286
Outliers excluded	484	510	567	470	513	527	543	536
Responses right	11287	11339	11331	11537	11450	11432	11476	11231
Responses left	11467	11404	11360	11571	11411	11461	11422	11055
Mean RT right (ms)	597.31	597.99	598.76	585.07	601.71	592.47	585.63	611.29
Mean RT left (ms)	589.71	591.88	609.62	581.68	603.56	606.95	591.54	634.56
Mean RT (left-right) (ms)	7.59	6.11	−10.86	3.38	−1.85	−14.47	−5.91	−23.26
Error Rate	0.0382	0.0375	0.0373	0.0241	0.0325	0.0306	0.0298	0.0554

In order to capture sequential effects in SNARC its slope was calculated separately as a function of the previous congruency history going back four trials to *t*−4, not including the current one at *t*. So for instance, in order to calculate the slop for the sequence CCCI, the SNARC slope was calculated for the subset of trials preceded by that sequence. Data for all sequences was plotted in a manner similar to that which is traditional in the sequential effects literature in order to facilitate comparison with previous results. The choice of five trials is based on the history of the field and because it provides a sufficiently distant horizon, i.e. events beyond the last five having usually little influence on results^21^.

### Reaction times as a function of response history

This was a classical sequential effects analysis where reaction times were grouped according to the history of responses going back four trials, including the trial being responded to. There are 32 possible five-long sequences of left/right responses, but these are grouped two by two and coded according to the pattern of repetitions and alternations. So for instance, denoting the two possible stimuli as X and Y, the sequences XYYXX and YXXYY would be written as ARAR, where A stands for an alternation and R for a repetition of the stimuli. The mean RT for all sequences of the same type across all participants was then calculated, excluding trials in which a mistake was made. Outliers beyond 2.5 standard deviations of the log transformed RTs were excluded from the analysis^28^.

## Results

We will discuss three main types of analysis: in the first we show the slope of the SNARC effect the classical way and how it varies with (1) the congruency of the last trial, (2) whether the current trial is a repetition or alternation (of the stimulus or of the response) and (3) the effect of the congruency of the last trial when restricting to response/stimulus repetitions/alternations. In the second type of analysis we look at the sequential effects profile of the slope of the SNARC effect, i.e. across all possible C/I histories, and how this profile varies if we restrict to stimulus/response alternations/repetitions. Finally, we will look at traditional sequential effects observed in our 2AFC task, i.e. the RT profile as a function of the history of responses.

### Classical analysis of the SNARC effect

**Figure 2:**
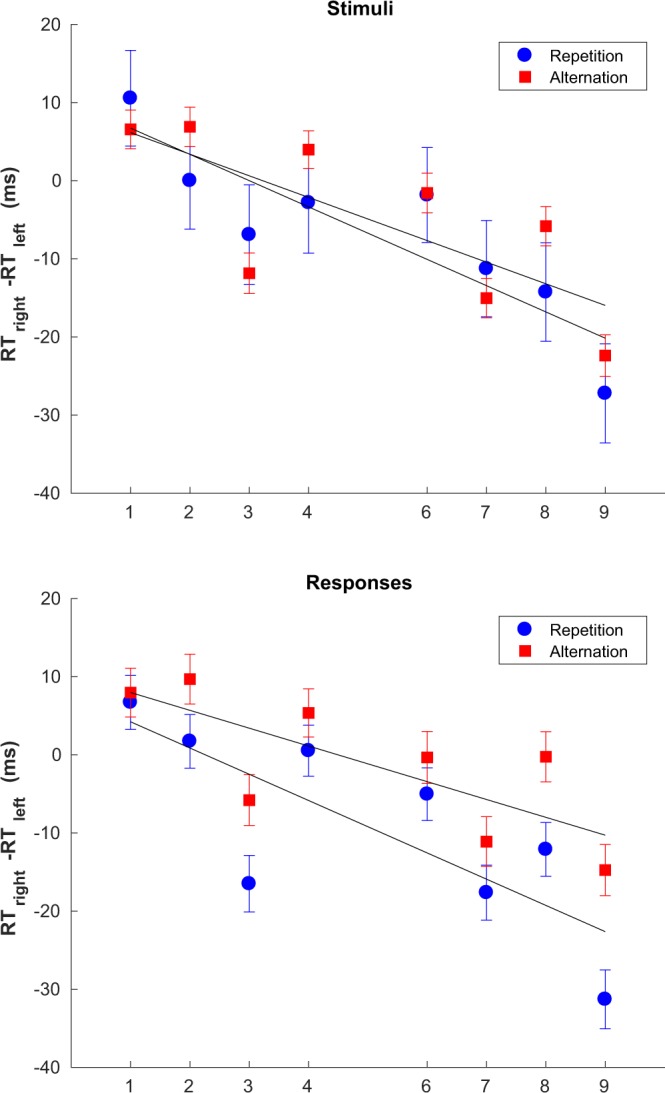
Effect of repetitions and alternations. Best fitting parameters: stimulus repetitions *b* = 10.09, *a* = −3.36 (*CI*_*α*=0.05_ = [−4.83, −1.88]); stimulus alternations *b* = 8.92, *a* = −2.76, (*CI*_*α*=0.05_ = [−4.72, −0.80]); response repetitions *b* = 7.58, *a* = −3.35, (*CI*_*α*=0.05_ = [−5.49, −1.21]); response alternations *b* = 10.25, *a* = −2.28, (*CI*_*α*=0.05_ = [−3.87, −0.69]). Error bars show the standard error of the difference between means.

As expected, we observed a significant overall SNARC effect in our data (see Fig. 1 top panel). Notably, we also observed an inversion of the SNARC effect if the last trial was incongruent (see Fig. 1 bottom panel). Finally, we studied the dependence of SNARC on whether the responses or the stimuli alternated or repeated between the current and the last trial (see Fig. 2). Our results indicate that, both in the case of responses and of stimuli, there is little difference in the slope of the SNARC effect between alternating and repeating trials.

**Figure 3:**
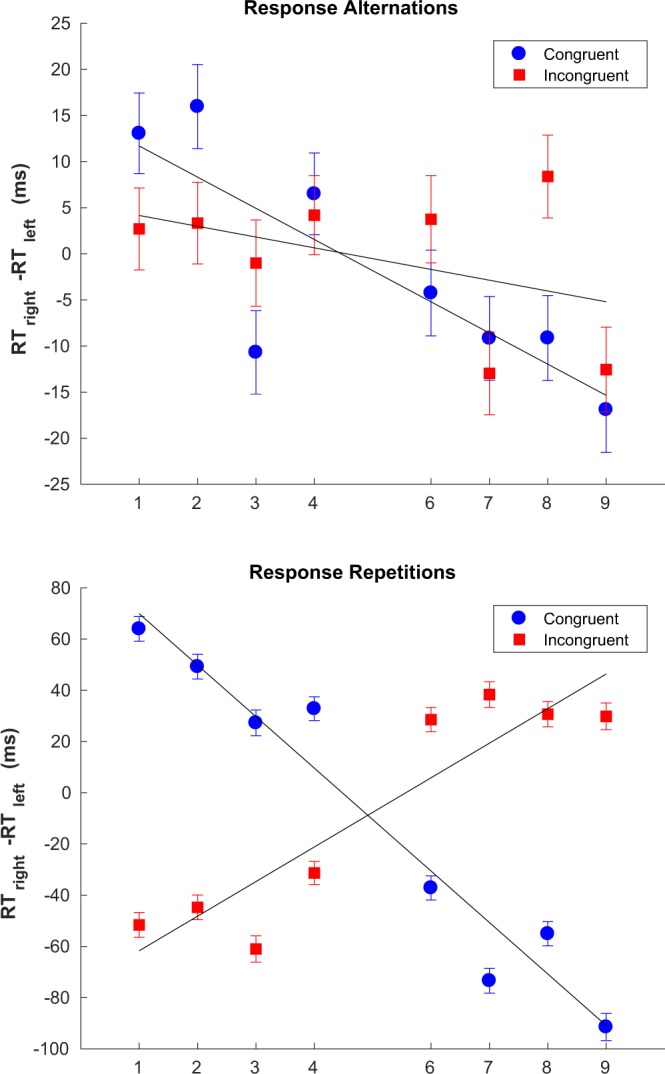
Modulation of the SNARC effect slope as a function of the congruency of the last trial for response alternations (top panel) and repetitions (bottom panel). Best fitting parameters: response alternations (incongruent last trial) *b* = 5.33, *a* = −1.17 (*CI*_*α*=0.05_ = [−3.13,0.79]); response alternations (congruent last trial) *b* = 15.08, *a* = −3.38, (*CI*_*α*=0.05_ = [−5.28, −1.47]); response repetitions (incongruent last trial); *b* = −75.21, *a* = 13.50 (*CI*_*α*=0.05_ = [8.81,18.19]); response repetitions (congruent last trial) *b* = 90, *a* = −20.09 (*CI*_*α*=0.05_ = [−23.94, −16.25]). Error bars show the standard error of the difference between means.

Figure 3 shows the modulation of the SNARC effect by the congruency of the last trial, separately for response alternations and repetitions. In the case of response alternations, an incongruent trial on the previous time step reduces the slope of the SNARC effect considerably, but does not invert it (Fig. 3, top panel). In other words, we still have a ‘normal’ SNARC effect whether the last trial was congruent or not. For response repetitions there is an inversion of the SNARC if the last trial was incongruent; if it was congruent, we continue to observe a SNARC effect with the usual slope (Fig. 3, bottom panel). Moreover, restricting to response repetitions considerably increases the absolute value of the slope whether the last trial was congruent or not.

**Figure 4:**
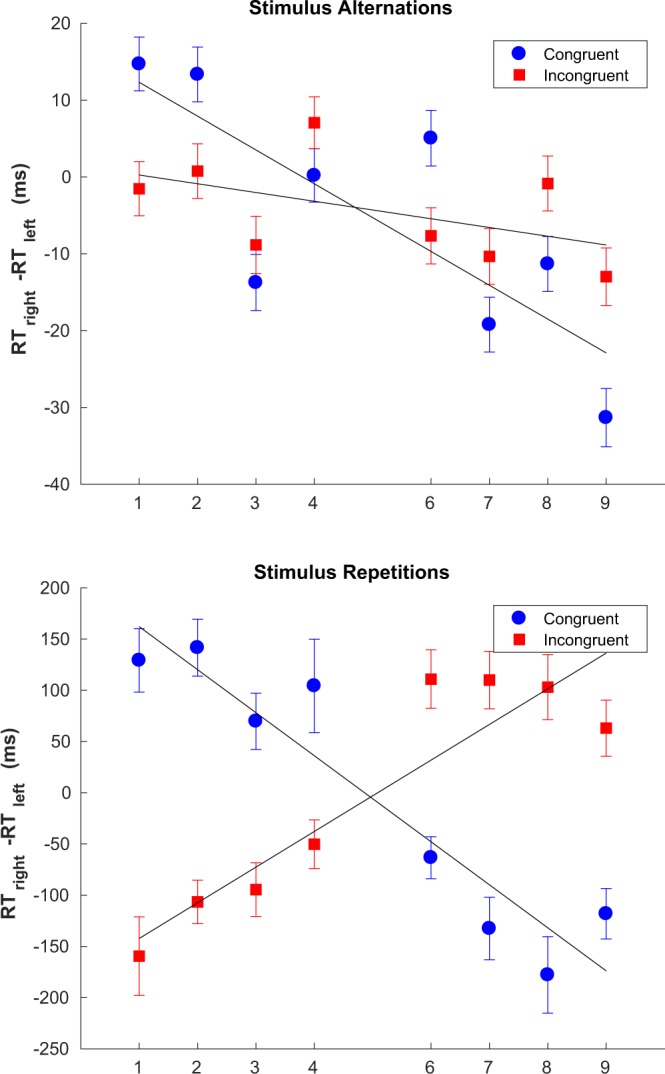
Modulation of the SNARC effect slope as a function of the congruency of the last trial for stimulus alternations (top panel) and repetitions (bottom panel). Best fitting parameters: stimulus alternations (incongruent last trial) *b* = 1.39, *a* = −1.1382 (*CI*_*α*=0.05_ = [−2.73,0.46]); stimulus alternations (congruent last trial) *b* = 16.72, *a* = −4.40 (*CI*_*α*=0.05_ = [−7.13, −1.66]); stimulus repetitions (incongruent last trial) *b* = −177.07, *a* = 34.81 (*CI*_*α*=0.05_ = [22.41,47.22]); stimulus repetitions (congruent last trial) *b* = 204.11, *a* = −41.99 (*CI*_*α*=0.05_ = [−54.02, −29.97]). Error bars show the standard error of the difference between means.

The way in which SNARC depends on the congruency of the last trial for stimulus repetitions and alternations - see Fig. 4 - is very similar to the case of repetitions. The only difference is that the absolute value of the slope when restricting to stimulus repetitions is even greater than for response repetitions (contrast the bottom panels of Figs 3 and 4).

### Sequential effects analysis of SNARC

**Figure 5:**
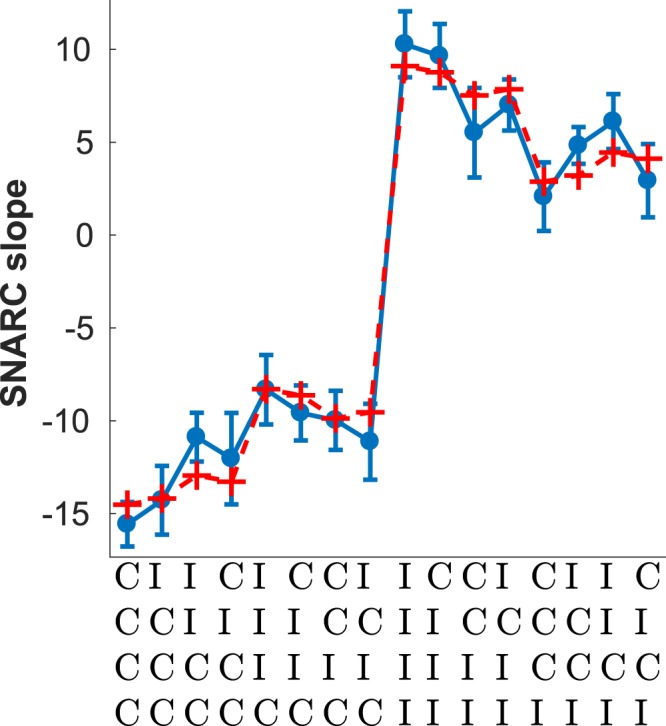
Sequential effects in SNARC. Blue line - Slope of the SNARC measured as a the best fitting linear regression of *RT*_*left*_ − *RT*_*right*_, mean response times with the left and right hands respectively, for each of the numbers (1, 2, 3, 4, 6, 7, 8, 9) displayed during the experiment. Red dashed line - best fitting exponential filter with *α* = 0.26, *a* = 9.1 and *b* = −23.6. *R*^2^ = 0.98. Error bars show the standard error of the mean.

Figure 5 shows the slope of the SNARC effect conditional on the history of congruent and incongruent trials, specifically the four trials preceding - but not including - the current one. Superimposed on the empirical data is the best fitting exponential filter of the sequence. The main feature of the data is the considerable difference in the slope of the SNARC effect depending on whether the last trial was congruent or not, visible as the average difference between the left and right halves of the plot. Crucially, when the last trial was C the slope of the SNARC effects is negative, whereas when it was an I it is positive. Note that a negative slope implies the traditional SNARC effect where RTs are quicker with the left hand to smaller numbers and with the right hand to larger numbers; conversely, a positive slope means that subjects were reacting faster with the right hand to smaller numbers and with the left hand to larger numbers. Overall, the dependence of the SNARC slope on the last four trials was well fit by an exponential filter of the sequence (*R*^2^ = 0.98). The last trial, and whether it is a C or an I, dominates the profile of sequential effects with the influence of trials beyond the last being relatively weak. This is reflected in the best fitting value of the memory parameter (*α* = 0.26) indicative of a relatively short memory.

**Figure 6:**
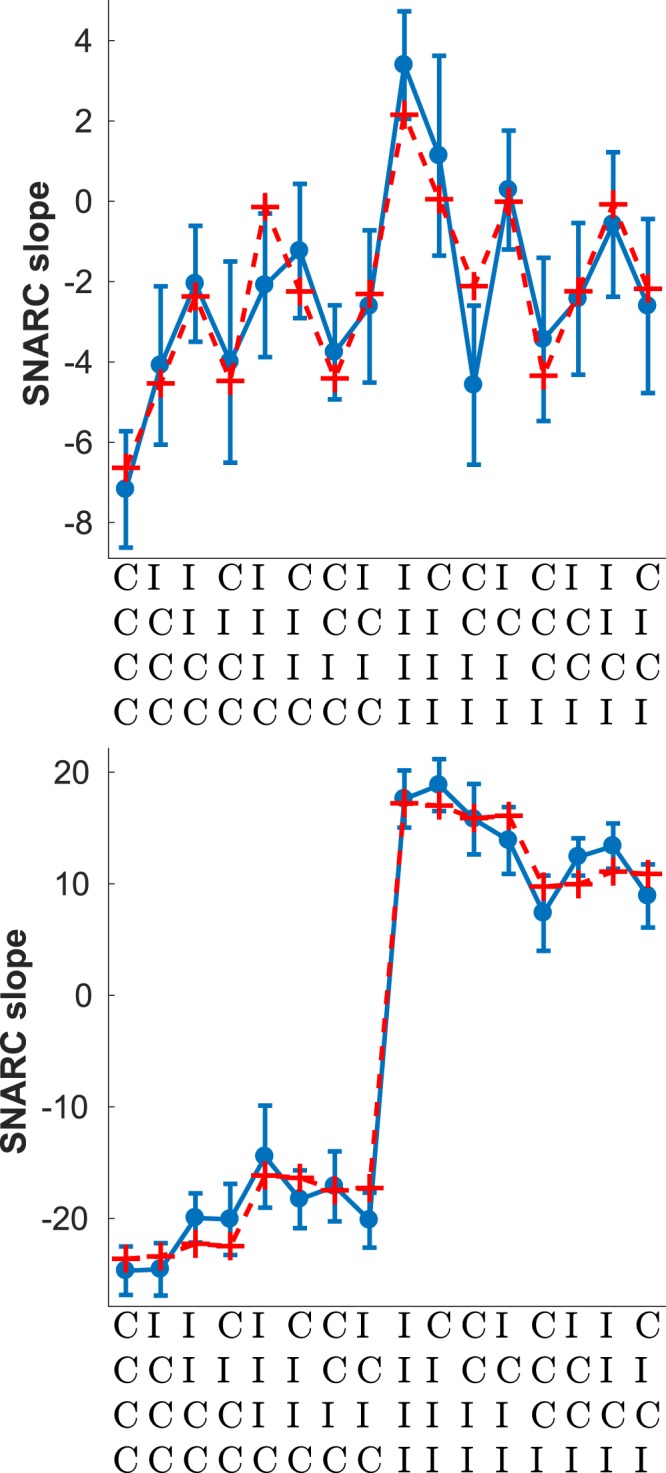
Sequential effects for response alternations (top) *α* = 0.97, *a* = −8.79 and *b* = 2.15, *R*^2^ = 0.86; and repetitions (bottom) *α* = 0.18, *a* = −40.8 and *b* = 17.2, *R*^2^ = 0.98. Error bars show the standard error of the mean.

**Figure 7:**
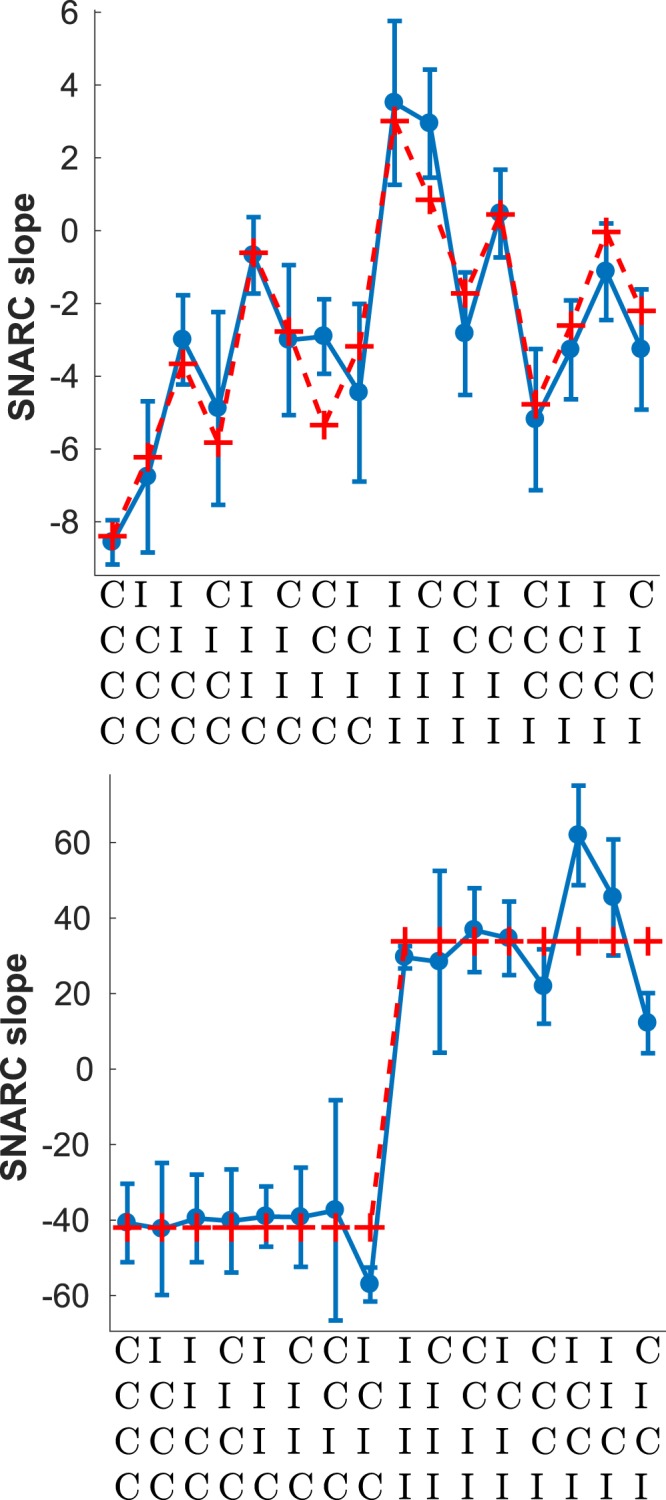
Sequential effects for stimulus alternations (top) *α* = 0.84, *a* = −11.4 and *b* = 3.01, *R*^2^ = 0.88; and repetitions (bottom) *α* ≈ 0, *a* = −75.7 and *b* = 33.8, *R*^2^ = 0.94. Error bars show the standard error of the mean.

The effect of response repetitions and alternations - shown in Fig. 6 - is similar to the stimulus case. Both repetitions and alternations have the same probability of occurrence in this case and so the data is split evenly between both cases. Unlike what was observed for stimulus repetitions, in the case of response repetitions there is still a hint of dependence on events further back than the last one, together with a lesser magnitude of the SNARC effect (or inverse SNARC if the last event was I). This is reflected in the best fitting value of *α* for response repetitions (Fig. 6, lower panel) when compared to stimulus repetitions (Fig. 7, lower panel), which is lower for response repetitions.

Figure 7 shows the profile of sequential effects in SNARC separately for stimulus repetitions and for alternations, the latter being defined in this case as two consecutive different stimuli. Stimulus repetitions are relatively rare compared to alternations since there are eight stimuli - 12.5% on average - resulting in an uneven partition of the data shown in Fig. 7. In both cases the best fitting exponential filter is shown together with the empirical data. Note the difference in absolute magnitude between the two plots: the SNARC effect is much stronger (either way) in the case of stimulus repetitions. Moreover, the overall SNARC effect - approximately equal to the mean of all 16 sequences - is close to 0 for stimulus repetitions, indicating the absence of the effect; conversely, in the case of stimulus alternations, a strongly negative SNARC effect is present. Finally, while there are strong sequential effects in the case of stimulus alternations, in the case of stimulus repetitions the effects of the sequence seem to reduce to a trivial dependence on the last trial and whether it was C or I. This is reflected in the best fitting value of *α* which is considerably higher for stimulus repetitions. Note that a high value of *α* implies a steep exponential decline into the past of the influence previous trials have on the probability assigned to the next trial, and in the limit of a very high *α* the exponential filter reduces to a trivial dependence on the last trial; this was the case for stimulus repetitions.

### Sequential effects in RT as a function of response history

**Figure 8:**
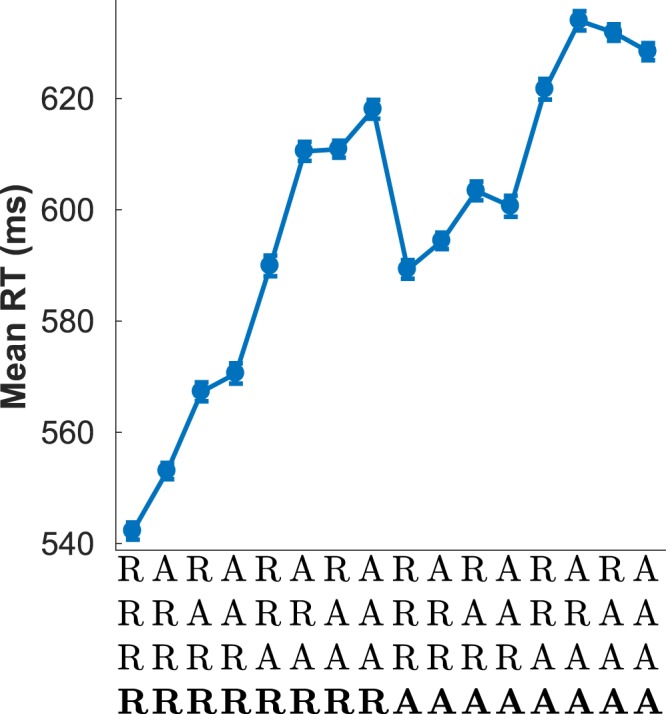
Mean reaction time as a function of the recent history of responses, coded as repetitions (R) and alternations (A) of the two possible responses (left and right). Sequences should be read from top to bottom with the current event - the one being responded to - shown in bold. The four-long A/R sequences therefore represent five-long sequences from *t* − 4 to *t* (the current stimulus). Error bars show the standard error of the mean.

Figure 8 shows mean RT as a function of the history of the last five responses, demonstrating that, concomitantly with sequential effects in SNARC, there were also substantial sequential effects as a function of the sequence of responses *per se*, as expected in a 2AFC. This profile of sequential effects is unusual and is thought to be associated with processing delays, as discussed below.

## Discussion

This work adds to the list of classical psychological effects that can be replicated by means of online crowdsourced data^27^. This is particularly striking as SNARC relies on subtle differences in reaction time, and reaction times collected online depend on different operating systems, browsers and keyboards, to name a few factors. However, online data collection allows for much higher numbers of participants which compensates for the extra noise.

Sequential effects in SNARC have been reported before, albeit only as a function of the immediately preceding trial and whether that trial was congruent or not. Pfister *et al*.^9^ report a reduced SNARC effect after an incongruent trial, and further that this reduction was stronger for response repetitions when compared to response alternations. Our results similarly show that the modulation of the SNARC effect by the history of congruency is notably stronger for response repetitions than for alternations. However, the results shown here differ from those of Pfister *et al*.^9^ in that we observe a significant *inversion* of the SNARC effect, rather than just a reduction, both for the overall results (Fig. 1, bottom panel) as well as when restricting to response repetitions (Fig. 3, bottom panel). When restricting to response alternations we observe a reduction in the SNARC effect but not an inversion (Fig. 3, top panel). While the general pattern (i.e. reduction) was the same in both studies, the lack of an inversion in Pfister *et al*.^9^ could lie with experimental differences. For example, Pfister *et al*. reinforced the spatial connotation using repeated framing instructions. Instructions are known to modulate spatial-numerical associations (e.g. Bachtold *et al*.^6^). It seems possible that these instructions might have strengthened the overall SNARC effect and hence weakened any sequential effects.

Tan *et al*.^8^ report that the SNARC effect is abolished for stimulus repetitions and for this reason we decided to investigate the effect of restricting to stimulus alternations (as well repetitions) on SNARC. Unlike Tan and Dixon^8^, we observed no difference in the magnitude of the SNARC effect between stimulus repetitions and alternations (see Fig. 2, top panel). For the sake of completion we repeated the same analysis for responses and found that, much like the case of stimuli, there was no significant overall difference in the magnitude of SNARC between response repetitions and alternations. We do not know the reason for these differences but point out that our results are potentially more reliable: Tan and Dixon^8^ ran 20 participants, each performing 500 trials, for a total of 10000 usable trials at best (by contrast with around 193000 trials in our experiment); since the probability of stimulus alternations for 8 stimuli is 12.5%, Tan *et al*.^8^ relied at best on 1250 trials for their conclusions with respect to stimulus repetitions, which would have to be further subdivided into 8 categories - one for each number - in order to calculate the slope. Another possible explanation for the differences observed is the response-stimulus interval used, which was 1000 ms in our study and 500 ms in the study by Tan and Dixon. The response-stimulus interval is known to influence the profile of sequential effects, which in turn could influence the way SNARC depends on repetitions and alternations of stimuli.

The differences in the way the SNARC effect depends on the congruency of the last trial can now be seen to be a part of wider changes in the overall profile of sequential effects as a function of the history of congruency beyond just the last trial (see Figs 6 and 7). In particular, and when restricting to stimulus or response alternations, there is a marked dependence on the congruency of trials beyond the last last one. When restricting to response repetitions the relative weight of the last event becomes greatly increased, and by contrast the effect of events beyond the last trial becomes apparently reduced, although not entirely absent in the case of response repetitions (see Fig. 6, bottom panel). In the case of stimulus repetitions there seems to be an almost ‘two-tiered’ dependence on the congruency of the last trial (see Fig. 7, bottom panel) but this result should be taken with caution given the relatively small number of stimulus alternations. We consider it to be more likely that the relatively small effects of the sequence beyond the last trial are masked by noise given the smaller amount of data.

The dependency of the SNARC effect on the congruency of the last trial changes markedly depending on whether there was an alternation or repetition of either the current response or the current stimulus (since the results are entirely analogous we will henceforth refer simply to repetitions or alternations). Repetitions are known to provide a facilitating effect in some circumstances, decreasing the overall reaction time relative to alternations^11^, and indeed reaction times for response repetitions were shorter than for alternations (contrast the left and right halves of Fig. 8). However, assuming the effect of repetitions (relative to alternations) was to linearly decrease RTs, we should not expect to see a change in the slope of SNARC. Therefore, the effect of repetitions on the slope of SNARC is not merely an artefact of restricting our analysis to a subset of trials - i.e. repetitions - with quicker RTs. Overall, this result indicates that there is some form of interference between the dependence of SNARC on the history of congruency and its dependence on the history of responses, with either repetitions facilitating SNARC or, conversely, alternations suppressing it.

### Sequential effects in reaction time

The pattern of results shown in Fig. 8 is known from the literature to be characteristic of 2AFC tasks with a very short response-stimulus interval (RSI)^29,30^ and is thought to be associated with a response processing conflict in situations where there are delays in processing due to ageing or very high task demands^20,29,30^. This is a somewhat surprising finding as our experiment made use of a 1000 ms RSI, whereas profiles similar Fig. 8 are usually elicited in simple 2AFCs by using an RSI of 50 ms^17,29^. The reason why this profile is observed with such a long interval between stimuli probably lies with the considerable processing demands of the SNARC task, in which subjects have to identify a number and make a parity judgement before responding. Nevertheless, our results stand as the first demonstration that the particular profile of sequential effects in reaction time shown in Fig. 8 can occur in a task with a long interval between trials.

## General Discussion

We have shown that the magnitude of the SNARC effects depends on the congruency of past trials beyond just the last one. Crucially, the way in which SNARC depends on the history of congruency is very similar to the way in which several other behavioural measures - such as RT, error rates and point of subjective equality - depend on the history of stimuli and/or responses in simple 2FACs. However, in this case the dependent measure is the magnitude of the SNARC effect, measured as the slope of the correlation between reaction time and number, which is reflective of a higher order cognitive effect. In addition, the sequence of events is itself higher order, consisting of whether a trial is congruent or not. The fact that the same type of sequential effects - well described by an exponential filter - can be found in such a wide range of tasks points to some form of universal underlying mechanism, possibly related to the detection of patterns in all types of sequence.

Sequential effects are thought to be the product of two components, one related to the detection of repetitions and another related to the detection of alternations^13,20^. In this context, the exponential filter effectively acts as a detector of repetitions: the predictions made by the filter are a simple function of how many events of a given type are present in the previous sequence and how recently they occurred. However, humans are usually also sensitive to alternating patterns, and so full sequential effects models are often more complex in order to account for this sensitivity^22,24^. Our results here indicate that sequential effects in SNARC can be explained by a simple exponential filter alone, and this means that SNARC is only sensitive to repetitions of either congruent or incongruent trials.

Unlike previous researchers we found that the SNARC effect inverts - rather than merely attenuates - when the last trial is incongruent. Such a discrepancy is also observed in the closely related Simon effect: Hommel *et al*.^31^ performed a series of experiments in order to assess how the Simon effect depended on the congruency of the last trial and found that in some experiments the effect inverted and others not. In both cases - SNARC and Simon - the discrepancies could be due to differences in experimental design but could also be due to individual differences. It is in fact known that there are strong individual differences in sequential effects in RT^20^. If such individual differences were also found in sequential effects in the SNARC and Simon effects it is possible that, for small sets of individuals, there would be considerable differences in whether the SNARC or Simon effects inverted as a function of the last trial. Unfortunately, the small amount of data usually collected per individual does not allow for clear conclusions to be drawn in this respect. However, the large number of subjects used here means that our results are more likely to be representative of the population as a whole, and that on average humans present an inverted SNARC effect if the last trial was incongruent.

The present findings add to the view that situated components can have a strong impact on spatial-numerical associations^32^. Situated magnitude processing (as opposed to grounded and embodied numerical cognition) contributes to the malleability of number-space associations. For instance, the SNARC effect can be inverted when stimulus numbers are conceived as hours on a clockface^6^. Also, placement of stimulus numbers in a line of text (toward the line’s left or right end) critically influence the direction of a SNARC effect^5^. Likewise, as shown by the present research, the placement of a number within long sequences of trials appears similarly decisive. Previous work has shown that repetition of a stimulus number may abolish SNARC^8^, but this kind of sequential effect was only investigated for a 2-trial lag^9^. It was recently pointed out^33^ that the early literature on sequence effects maintained that the influence on previous trials is restricted to the two immediately preceding trials^34,35^. Were this the case, an influential theoretical model of the SNARC effect - the working memory account of number-space associations^36^ - would not be valid. These authors proposed that (in)compatibility effects arise from participants’ working memory. The results of our experiment clearly show that a trial’s influence will not decay after only one or two trials and is therefore an important finding in support of the working memory view of spatial-numerical associations^33,37^.

The modulation of SNARC on a trial-by-trial basis, and in particular the fact that it can be inverted, shows that SNARC is a dynamic part of the decision process. In other words, humans are constantly attempting to optimise their decision process and this includes deciding whether they should associate large numbers with right responses or with left responses, and vice-verse for small numbers. The fact that overall humans display a preference for associating large numbers with right responses may reflect a prior belief that that is the correct mapping, possibly based on the prevalence of left-to-right number and reading systems. However, our work shows that this prior belief can be quickly overriden by a single incongruent trial, demonstrating the flexibility of SNARC.
